# Greedy caliper propensity score matching can yield variable estimates of the treatment‐outcome association—A simulation study

**DOI:** 10.1002/pds.5232

**Published:** 2021-03-25

**Authors:** Joris J. Komen, Svetlana V. Belitser, Richard Wyss, Sebastian Schneeweiss, Anne C. Taams, Romin Pajouheshnia, Tomas Forslund, Olaf H. Klungel

**Affiliations:** ^1^ Division of Pharmacoepidemiology and Clinical Pharmacology Utrecht Institute of Pharmaceutical Sciences, Utrecht University Utrecht The Netherlands; ^2^ Department of Healthcare Development Stockholm County Council, Public Healthcare Services Committee Stockholm Sweden; ^3^ Division of Pharmacoepidemiology and Pharmacoeconomics, Department of Medicine Brigham and Women's Hospital and Harvard Medical School Boston Massachusetts USA; ^4^ Department of Medicine Solna, Clinical Epidemiology/Clinical Pharmacology Karolinska Institutet Stockholm Sweden

**Keywords:** greedy, matching, caliper matching, matching, nearest neighbor matching, pharmacoepidemiology, propensity score, simulation

## Abstract

**Purpose:**

Greedy caliper propensity score (PS) matching is dependent on randomness, which can ultimately affect causal estimates. We sought to investigate the variation introduced by this randomness.

**Methods:**

Based on a literature search to define the simulation parameters, we simulated 36 cohorts of different sizes, treatment prevalence, outcome prevalence, treatment‐outcome‐association. We performed 1:1 caliper and nearest neighbor (NN) caliper PS‐matching and repeated this 1000 times in the same cohort, before calculating the treatment‐outcome association.

**Results:**

Repeating caliper and NN caliper matching in the same cohort yielded large variations in effect estimates, in all 36 scenarios, with both types of matching. The largest variation was found in smaller cohorts, where the odds ratio (OR) ranged from 0.53 to 10.00 (IQR of ORs: 1.11‐1.67). The 95% confidence interval was not consistently overlapping a neutral association after repeating the matching with both algorithms. We confirmed these findings in a noninterventional example study.

**Conclusion:**

Caliper PS‐matching can yield highly variable estimates of the treatment‐outcome association if the analysis is repeated.

**Key Points**

- Greedy caliper propensity score matching is the most frequently used matching algorithm.
- Repeating greedy caliper propensity score matching in the same cohort yielded high variation in the treatment‐outcome association.
- The confidence interval of the treatment‐outcome association did not consistently overlap a neutral association after repeating the matching procedure in the same cohort.
- This variation was largest in cohorts with lower outcome prevalence, in which propensity score matching is frequently used.
- Our findings were confirmed in a real‐life pharmacoepidemiologic study, comparing two antithrombotic treatments and the risk of stroke in patients with atrial fibrillation.

## 1 BACKGROUND

In observational research, treatment allocation is not random, but allocated by the treating physician. Therefore, patient characteristics will likely influence the physician's decision to give a patient a certain treatment, or not^1^. Adjusting for these characteristics can decrease this bias, and the propensity score (PS) is often used for this purpose^2^. Besides using the PS for adjustment, weighting, or stratification^3^, using the PS for matching is a popular way to achieve cohorts with comparable baseline characteristics^4^, ^5^.

Greedy caliper matching is a popular method used in PS matching^6^. This method orders the treated subjects, and the first treated subject is randomly matched to an untreated (or alternatively treated) subject with a PS that is within a predefined caliper width. The initial ordering of subjects is often done randomly but may also be based on a subject's PS or other parameters. In addition to caliper matching, nearest neighbor (NN) caliper matching is often used, where the treated subject is matched to an untreated subject that has the closest propensity score within the caliper. Both methods do not consider that the untreated subject can potentially form a better pair with another treated subject that is further down the line; hence they are “greedy” algorithms. Because of this, both methods are dependent on the random order in which the treated subjects are placed, if patients are not ordered based on their PS. In addition, caliper matching is also dependent on which untreated patient within the caliper is randomly matched.

Most statistical programs use a pseudo‐random ordering, which can allow for the random ordering to be reproduced if the same random seed is used. However, how much the matching, and ultimately the estimated treatment effect, can differ with a different random seed, is unknown. We evaluated the extent to which observational studies analyzed using greedy caliper PS matching with random ordering and greedy NN caliper PS matching are susceptible to variable results due to the randomness in the matching.

## 2 METHODS

The study consisted of three parts. First, we conducted a review of matching procedures used in epidemiologic studies to identify realistic scenarios for a simulation study. Second, we repeatedly applied PS matching in several simulated cohorts. Third, we sought to replicate the findings in a real observational study of drug effectiveness.

### 2.1 Literature search

We performed a literature search to find realistic parameters for our simulation. In PubMed, we searched for “propensity score” AND (([match] OR matched) OR matching), filtering core clinical journals as defined by PubMed. The search was performed on August 22, 2019. We selected the 50 most recently published pharmacoepidemiology studies using PS matching, and 50 studies that were not pharmacoepidemiology, as defined by two independent reviewers (J.K. and A.T.). From these articles, we identified the matching algorithm that was used, which statistical program was used, the sample size, the treatment prevalence, the outcome prevalence, and the strength of the association between treatment and outcome. These parameters were used to determine the parameters of the simulation study.

### 2.2 Data simulation

We simulated a range of cohorts based on scenarios identified through our literature search. We simulated cohorts of different sizes (500, 2500, 10 000), different treatment prevalence (20%, 50%), different outcome prevalence (10%, 50%), and different associations between treatment and outcome (OR of 0.75, 1.0, 1.5), yielding 36 scenarios.

For the simulation of the cohorts, we used a 2‐step process to define covariates. First, we created 8 variables (X_1_ ⋯X_8_): 6 binary variables (X_1_ ⋯X_6_) and 2 continuous variables (X_7_, X_8_). X_1_ through X_6_ were randomly drawn from a binomial distribution and had a prevalence of 0.2 and both X_7_ and X_8_ were drawn from a normal distribution and had a mean of 0 and a variance of 0.5 unit. All covariates were independent of each other. Based on these variables, we defined the probability of treatment *T* using a logistic model, and then simulated T from these probabilities:

$$p\left(T|X_{1}\cdots X_{8}\right)={\left(1+\exp\left(-\left(\alpha_{0}+\alpha_{1}X_{1}+\ldots +\alpha_{8}X_{8}\right)\right)\right)}^{-1}$$

Finally, we simulated outcome *Y* based on the probability of *Y* given all eight variables and the treatment *T*, using a logistic model:

$$p\left(Y|T,X_{1}\cdots X_{8}\right)={\left(1+\exp\left(-\left(\beta_{0}+\beta_{1}X_{1}+\ldots +\beta_{8}X_{8}+\beta_{T}T\right)\right)\right)}^{-1}$$

The range of values used in the models in different scenarios is presented in Table 1. The parameter values *α*_0_ and *β*_0_ were chosen to result in the desired prevalence for *T* of 0.2 and 0.5 and for *Y* of 0.1 and 0.5.

**TABLE 1:** Parameters for the simulation study and the corresponding values

Variable^a^	Prevalence/mean(var)^b^	OR_T_ ^c^	Parameter	OR_Y_ ^d^	Parameter
X_1_	0.2	2	α_1_	1	β_1_
X_2_	0.2	1	α_2_	2	β_2_
X_3_	0.2	0.5	α_3_	0.5	β_3_
X_4_	0.2	2	α_4_	0.5	β_4_
X_5_	0.2	1	α_5_	1	β_5_
X_6_	0.2	0.5	α_6_	2	β_6_
X_7_	0 (0.5)	1.5	α_7_	0.5	β_7_
X_8_	0 (0.5)	0.5	α_8_	1.5	β_8_
T	0.2, 0.5^e^		α_0_	0.75, 1.0, 1.5	β_9_
Y	0.1, 0.2^f^				β_0_

*Note:* Parameters were chosen based on the results from the literature review to create different scenarios with two levels of treatment prevalence, two levels of outcome prevalence, and three different treatment‐outcome associations.

^a^ Variable X_1_ through X_6_ are binary variables. Variable X_7_ and X_8_ are continuous variables.

^b^ Prevalence for all binary variables (X_1_ through X_2_) and mean with variance for all continuous variables (X_7_ and X_8_).

^c^ Odds ratio for the relation between parameter α and the treatment T, corresponding to formula 1.

^d^ Odds ratio for the relation between parameter β and the outcome Y, corresponding to formula 2.

^e^ Treatment prevalence of 20% and 50% in the whole population (approximate number).

^f^ Outcome prevalence of 10% and 20% in the whole population (approximate number).

### 2.3 Propensity score matching

In all 36 generated cohorts, we applied greedy caliper matching, with and without using NN. First, in all 36 cohorts, we used logistic regression to calculate the probability for the treatment based on the simulated covariates, which was used as the PS. Then we used both matching methods in a 1:1 fashion without replacement and with a random ordering of treated patients, as was most used in the literature search and which is the default option in most statistical packages. We varied the caliper width using 0.2 and 0.01 of the standard deviation of the propensity score (SD_ps_). In all 36 cohorts, we replicated both matching algorithms 1000 times with a different random seed for each repetition, to create a different order for each repetition. In all 1000 matched sets, we performed a conditional logistic regression for matched pairs, only including treatment and outcome, to calculate the association between treatment and outcome after matching. All statistical analyses were performed with statistical software R version 3.4.2 and RStudio Desktop version 1.1.463. We used a modification of the “MatchIt” package for the matching procedures^7^. That is, in the MatchIt package it is by default not possible to perform NN caliper matching, but only NN matching without calipers or caliper matching without NN. The modification allowed us to perform NN caliper matching.

We present the median, interquartile range (IQR), and full range of the 1000 ORs, coming from the corresponding 1000 matched sets. Second, we present the unadjusted OR in the full cohort. Third, we present the proportion of matched sets that yielded statistically significant results, both positive and negative (ie, 95% confidence interval of the OR not containing 1). Fourth, we present the proportion of matched sets that were unsuccessfully matched (ie, at least one of the covariates had a standardized mean difference [SMD] > 0.1 after PS matching). We performed a sensitivity analysis in which we excluded all unsuccessfully matched cohorts. Fifth, we present the mean number of matched subjects. We only present the results for the matching with a caliper width of 0.2 SD_ps_. The results after matching with a caliper width of 0.01 SD_ps_ can be found in the appendix.

### 2.4 Real‐life dataset

We used the Stockholm Healthcare database for confirmation of our findings from the simulation dataset in a real‐life setting. The database has been described elsewhere^8^. In short, the database contains demographic information for all Stockholm residents (n = 2.3 million), ATC‐codes for dispensed drugs, and ICD‐10 codes for inpatient and outpatient diagnoses from primary and secondary care.

From this database, we selected all patients prescribed with a vitamin K antagonist (VKA) or a non VKA oral anticoagulant (NOAC) with a prior diagnosis of atrial fibrillation (ICD‐10: I48) and no claim for any oral anticoagulant (OAC) in the year prior to inclusion. To vary the sample size of the cohort, we created the smallest cohort including patients initiated in the last quarter of 2013, a medium cohort including all patients initiated in 2013, and a large cohort of patients initiated in 2013 until 2015. The first prescription was defined as the index date and patients were followed for a maximum of one year. Patients were censored when they emigrated, died, or suffered from an outcome. The outcome of interest was a composite of an ischemic stroke, unspecified stroke or transient ischemic attack (TIA), registered as an ICD‐10 code in a hospital setting and requiring acute care, as was done previously^9^.

We used a PS matched intention‐to‐treat analysis to assess the association of NOACs versus VKA and the risk for the composite endpoint. The propensity score was the probability of receiving a NOAC compared to a VKA, calculated using logistic regression. In the logistic regression model, we used the components of the CHA_2_Ds_2_‐VASC score (age, sex, heart failure, hypertension, prior stroke/TIA/embolism, vascular disease, and diabetes), registered in the 5 years prior to index date^10^.

We used both 1:1 caliper matching and 1:1 NN caliper matching with a caliper width of 0.2 SD_ps_ or 0.01 SD_ps_ without replacement. We replicated the matching procedure 1000 times with a different random seed for each repetition. In each matched set, we used a stratified Cox proportional hazards model for matched pairs to assess the association of NOAC vs VKA with the risk for the composite outcome.

## 3 RESULTS

### 3.1 Literature search

We assessed 100 articles. Of the 72 articles mentioning the kind of matching algorithm used, 51 used nearest neighbor matching (32 with a caliper), 17 used caliper matching, two used 5:1‐digit matching, one used optimal matching, and one used kernel matching. SAS was mentioned in 32 articles, R in 25, SPSS in 17, and STATA in 14. The MatchIt package in R was the most frequently mentioned package (n = 13) but most often no package, macro, or program was mentioned at all (n = 79).

### 3.2 Simulation study

**FIGURE 1:**
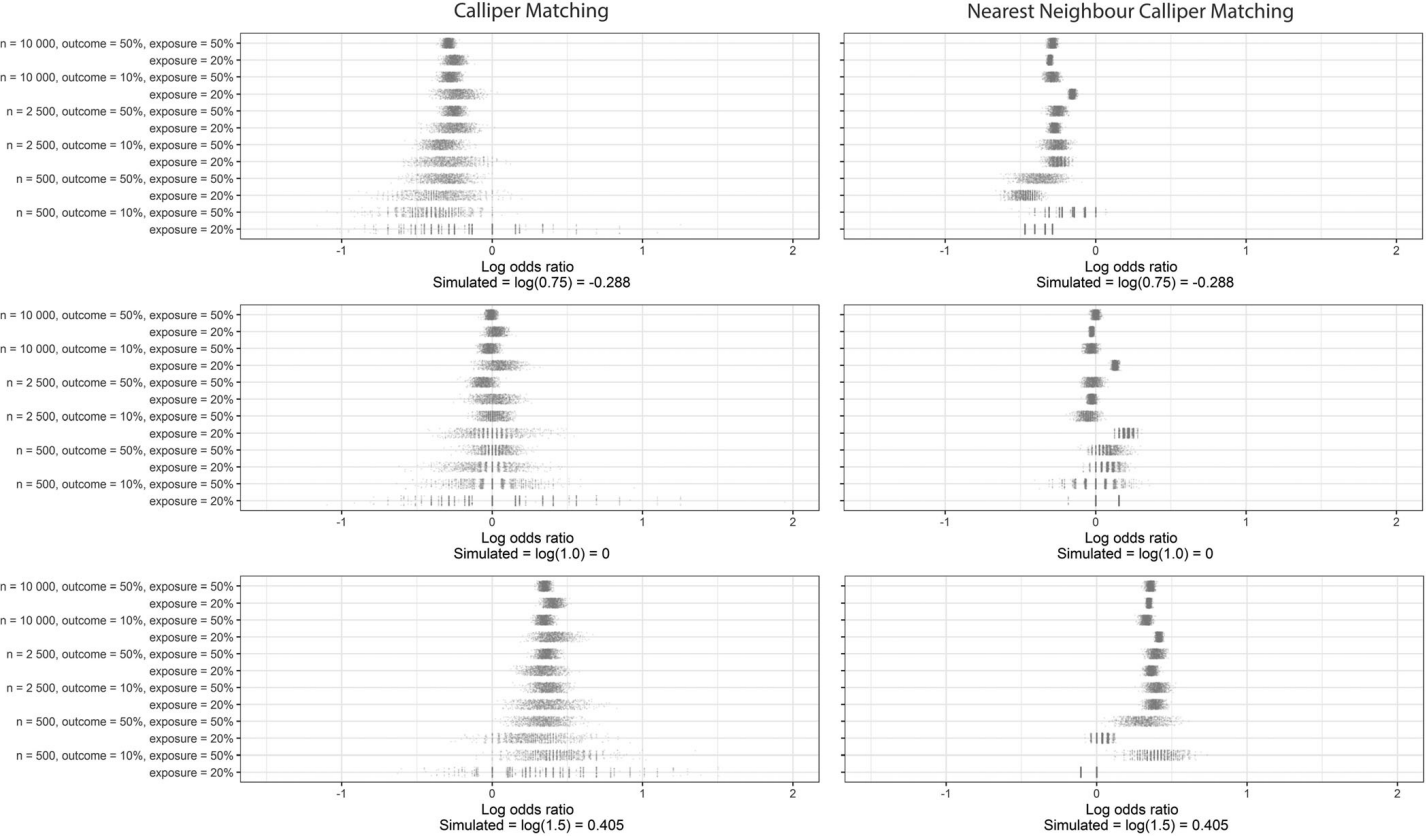
Scatterplot of the distribution of odds ratios in the 1000 matched sets after caliper matching and after nearest neighbor caliper matching in the different simulation scenarios

Repeating the PS matching 1000 times with a different random seed yielded wide variation in the OR for the association of treatment and outcome, especially in caliper matching and less in NN caliper matching (see Figure 1 and Table 2, 3, and 4; Table A1a‐c). The variation was largest with caliper matching in a sample size of 500, where the smallest OR was 0.53 (CI: 0.23‐1.26) and the largest was 10.00 (CI: 1.28‐78.1) with an IQR from 1.11 to 1.67.

**TABLE 2:** Results from the 1000 matched sets in different scenarios with a simulated odds ratio of 0.75 and a caliper width of 0.2 SD_ps_

		Median OR	Interquartile range OR	Full range OR	Unadjusted OR	% Sign low risk	% Sign high risk	Mean n matches	% Unsuccessful matches
N = 10.000									
Caliper matching	T 50%, O 50%	0.75	(0.74‐0.76)	(0.69‐0.81)	0.59	100.0%	0.0%	7266	0.0%
	T 20%, O 50%	0.78	(0.76‐0.80)	(0.70‐0.89)	0.58	99.9%	0.0%	3902	0.0%
	T 50%, O 10%	0.76	(0.74‐0.78)	(0.67‐0.84)	0.56	100.0%	0.0%	7049	0.0%
	T 20%, O 10%	0.79	(0.76‐0.83)	(0.66‐1.03)	0.54	45.4%	0.0%	3902	0.0%
Nearest neighbor caliper matching	T 50%, O 50%	0.75	(0.74‐0.76)	(0.71‐0.79)	0.59	100.0%	0.0%	7162	0.0%
	T 20%, O 50%	0.74	(0.73‐0.74)	(0.72‐0.76)	0.58	100.0%	0.0%	3898	0.0%
	T 50%, O 10%	0.75	(0.74‐0.76)	(0.70‐0.80)	0.56	100.0%	0.0%	6935	0.0%
	T 20%, O 10%	0.86	(0.85‐0.86)	(0.83‐0.89)	0.54	0.0%	0.0%	3898	0.0%
N = 2500									
Caliper matching	T 50%, O 50%	0.78	(0.76‐0.79)	(0.69‐0.86)	0.62	97.6%	0.0%	1883	0.0%
	T 20%, O 50%	0.78	(0.74‐0.82)	(0.61‐1.02)	0.59	49.4%	0.0%	1048	3.1%
	T 50%, O 10%	0.72	(0.69‐0.75)	(0.56‐0.90)	0.52	48.2%	0.0%	1883	0.0%
	T 20%, O 10%	0.74	(0.69‐0.81)	(0.55‐1.13)	0.65	10.9%	0.0%	1038	3.6%
Nearest neighbor caliper matching	T 50%, O 50%	0.78	(0.76‐0.79)	(0.70‐0.84)	0.62	99.6%	0.0%	1865	0.0%
	T 20%, O 50%	0.76	(0.75‐0.77)	(0.72‐0.82)	0.59	91.6%	0.0%	1046	0.0%
	T 50%, O 10%	0.77	(0.75‐0.79)	(0.67‐0.88)	0.52	3.0%	0.0%	1865	0.0%
	T 20%, O 10%	0.78	(0.76‐0.80)	(0.69‐0.88)	0.65	0.0%	0.0%	1031	0.0%
N = 500									
Caliper matching	T 50%, O 50%	0.73	(0.68‐0.78)	(0.51‐1.00)	0.53	10.5%	0.0%	339	37.4%
	T 20%, O 50%	0.71	(0.64‐0.79)	(0.39‐1.22)	0.54	6.9%	0.0%	210	71.9%
	T 50%, O 10%	0.67	(0.61‐0.75)	(0.33‐1.18)	0.55	1.5%	0.0%	353	25.4%
	T 20%, O 10%	0.78	(0.67‐1.00)	(0.31‐3.50)	0.60	0.2%	0.0%	202	76.2%
Nearest neighbor caliper matching	T 50%, O 50%	0.69	(0.65‐0.73)	(0.53‐0.91)	0.53	19.6%	0.0%	335	41.2%
	T 20%, O 50%	0.63	(0.60‐0.65)	(0.52‐0.73)	0.54	8.0%	0.0%	207	94.6%
	T 50%, O 10%	0.86	(0.79‐0.92)	(0.57‐1.08)	0.55	0.0%	0.0%	347	24.9%
	T 20%, O 10%	0.71	(0.67‐0.71)	(0.63‐0.75)	0.60	0.0%	0.0%	202	20.7%

*Note:* OR = odds ratio; T 50% = treatment prevalence 50%; O 50% = outcome prevalence 50%; % sign low/high risk = percentage of the 1000 matched sets with a significantly increased or decreased risk.

**TABLE 3:** Results from the 1000 matched sets in different scenarios with a simulated odds ratio of 1.0 and a caliper width of 0.2 SD_ps_

		Median OR	Interquartile range OR	Full range OR	Unadjusted OR	% Sign low risk	% Sign high risk	Mean n matches	% Unsuccessful matches
N = 10.000									
Caliper matching	T 50%, O 50%	0.99	(0.98‐1.01)	(0.91‐1.04)	0.77	0.0%	0.0%	7266	0.0%
	T 20%, O 50%	1.02	(1.00‐1.05)	(0.92‐1.17)	0.76	0.0%	0.2%	3902	0.0%
	T 50%, O 10%	0.98	(0.96‐1.00)	(0.90‐1.07)	0.74	0.0%	0.0%	7049	0.0%
	T 20%, O 10%	1.05	(1.01‐1.10)	(0.87‐1.37)	0.72	0.0%	0.5%	3902	0.0%
Nearest neighbor caliper matching	T 50%, O 50%	1.00	(0.99‐1.01)	(0.96‐1.05)	0.77	0.0%	0.0%	7162	0.0%
	T 20%, O 50%	0.97	(0.97‐0.98)	(0.95‐1.00)	0.76	0.0%	0.0%	3898	0.0%
	T 50%, O 10%	0.97	(0.96‐0.99)	(0.91‐1.04)	0.74	0.0%	0.0%	6935	0.0%
	T 20%, O 10%	1.14	(1.13‐1.15)	(1.09‐1.18)	0.72	0.0%	0.0%	3898	0.0%
N = 2500									
Caliper matching	T 50%, O 50%	0.94	(0.92‐0.97)	(0.79‐1.10)	0.77	0.2%	0.0%	1731	0.0%
	T 20%, O 50%	1.02	(0.96‐1.07)	(0.77‐1.30)	0.68	0.0%	0.0%	954	4.3%
	T 50%, O 10%	1.01	(0.98‐1.05)	(0.85‐1.17)	0.77	0.0%	0.0%	1823	0.0%
	T 20%, O 10%	1.00	(0.89‐1.10)	(0.65‐1.72)	0.63	0.0%	0.0%	911	8.0%
Nearest neighbor caliper matching	T 50%, O 50%	0.98	(0.96‐1.00)	(0.90‐1.08)	0.77	0.0%	0.0%	1701	0.0%
	T 20%, O 50%	0.97	(0.96‐0.98)	(0.91‐1.03)	0.68	0.0%	0.0%	949	0.0%
	T 50%, O 10%	0.95	(0.92‐0.98)	(0.82‐1.07)	0.77	0.0%	0.0%	1809	0.0%
	T 20%, O 10%	1.24	(1.21‐1.25)	(1.13‐1.36)	0.63	0.0%	0.0%	907	0.0%
N = 500									
Caliper matching	T 50%, O 50%	1.02	(0.96‐1.08)	(0.73‐1.35)	0.70	0.0%	0.0%	357	23.4%
	T 20%, O 50%	0.96	(0.87‐1.09)	(0.54‐1.59)	0.74	0.0%	0.0%	190	83.6%
	T 50%, O 10%	1.00	(0.88‐1.13)	(0.53‐2.57)	0.78	0.0%	0.1%	349	19.6%
	T 20%, O 10%	1.00	(0.78‐1.20)	(0.33‐7.00)	0.60	0.0%	0.0%	190	83.6%
Nearest neighbor caliper matching	T 50%, O 50%	1.08	(1.03‐1.13)	(0.89‐1.34)	0.70	0.0%	0.0%	356	53.1%
	T 20%, O 50%	1.08	(1.04‐1.13)	(0.92‐1.30)	0.74	0.0%	0.0%	190	81.9%
	T 50%, O 10%	1.00	(0.93‐1.07)	(0.67‐1.43)	0.78	0.0%	0.0%	346	9.0%
	T 20%, O 10%	1.17	(1.00‐1.17)	(0.83‐1.17)	0.60	0.0%	0.0%	190	81.9%

*Note:* OR = odds ratio; T 50% = treatment prevalence 50%; O 50% = outcome prevalence 50%; % sign low/high risk = percentage of the 1000 matched sets with a significantly increased or decreased risk.

**TABLE 4:** Results from the 1000 matched sets in different scenarios with a simulated odds ratio of 1.5 and a caliper width of 0.2 SD_ps_

		Median OR	Interquartile range OR	Full range OR	Unadjusted OR	% sign low risk	% sign high risk	Mean n matches	% unsuccessful matches
N = 10.000									
Caliper matching	T 50%, O 50%	1.42	(1.40‐1.44)	(1.33‐1.51)	1.11	0.0%	100.0%	7266	0.0%
	T 20%, O 50%	1.50	(1.46‐1.54)	(1.34‐1.69)	1.08	0.0%	100.0%	3902	0.0%
	T 50%, O 10%	1.41	(1.38‐1.44)	(1.29‐1.55)	1.05	0.0%	100.0%	7049	0.0%
	T 20%, O 10%	1.49	(1.43‐1.57)	(1.21‐1.95)	1.05	0.0%	99.8%	3820	0.0%
Nearest neighbor caliper matching	T 50%, O 50%	1.43	(1.41‐1.44)	(1.35‐1.50)	1.11	0.0%	100.0%	7162	0.0%
	T 20%, O 50%	1.42	(1.41‐1.42)	(1.38‐1.46)	1.08	0.0%	100.0%	3898	0.0%
	T 50%, O 10%	1.39	(1.37‐1.41)	(1.29‐1.48)	1.05	0.0%	100.0%	6935	0.0%
	T 20%, O 10%	1.51	(1.50‐1.53)	(1.46‐1.57)	1.05	0.0%	100.0%	3807	0.0%
N = 2500									
Caliper matching	T 50%, O 50%	1.43	(1.40‐1.47)	(1.26‐1.62)	1.04	0.0%	100.0%	1823	0.0%
	T 20%, O 50%	1.40	(1.34‐1.47)	(1.13‐1.79)	1.09	0.0%	89.4%	1038	3.6%
	T 50%, O 10%	1.45	(1.39‐1.51)	(1.15‐1.73)	1.01	0.0%	92.0%	1731	0.0%
	T 20%, O 10%	1.44	(1.33‐1.56)	(1.04‐2.29)	1.25	0.0%	37.9%	1038	3.6%
Nearest neighbor caliper matching	T 50%, O 50%	1.48	(1.46‐1.51)	(1.35‐1.61)	1.04	0.0%	100.0%	1809	0.0%
	T 20%, O 50%	1.44	(1.42‐1.46)	(1.34‐1.55)	1.09	0.0%	100.0%	1031	0.0%
	T 50%, O 10%	1.49	(1.45‐1.53)	(1.29‐1.69)	1.01	0.0%	99.8%	1701	0.0%
	T 20%, O 10%	1.47	(1.44‐1.50)	(1.33‐1.64)	1.25	0.0%	38.2%	1031	0.0%
N = 500									
Caliper matching	T 50%, O 50%	1.41	(1.31‐1.50)	(1.00‐2.04)	1.09	0.0%	13.4%	326	27.0%
	T 20%, O 50%	1.30	(1.17‐1.47)	(0.76‐2.50)	1.05	0.0%	2.7%	187	82.6%
	T 50%, O 10%	1.50	(1.35‐1.69)	(0.90‐3.86)	1.17	0.0%	4.0%	349	19.6%
	T 20%, O 10%	1.29	(1.11‐1.67)	(0.53–10.00)	0.96	0.0%	0.4%	185	87.7%
Nearest neighbor caliper matching	T 50%, O 50%	1.37	(1.29‐1.45)	(1.03‐1.85)	1.09	0.0%	4.0%	322	32.6%
	T 20%, O 50%	1.04	(1.00‐1.07)	(0.92‐1.14)	1.05	0.0%	0.0%	187	87.6%
	T 50%, O 10%	1.50	(1.40‐1.60)	(1.07‐2.08)	1.17	0.0%	0.4%	346	9.0%
	T 20%, O 10%	1.00	(0.90‐1.00)	(0.90‐1.00)	0.96	0.0%	0.0%	183	100.0%

*Note:* OR = odds ratio; T 50% = treatment prevalence 50%; O 50% = outcome prevalence 50%; % sign low/high risk = percentage of the 1000 matched sets with a significantly increased or decreased risk.

Originating from the same cohort, some matched sets yielded a 95% confidence interval that overlapped 1, while other matched sets did not, both after applying caliper matching as in NN caliper matching. For example, in a cohort with a simulated OR of 1.5 (n = 2500), in 37.9% of the cases after caliper matching and in 38.2% of the cases after NN caliper matching, the 95% confidence interval did not overlap 1, while in the other cases it did.

When only including successfully matched sets, i.e., only sets with all covariates having a SMD ≤ 0.1 after matching, the variation was smaller for both caliper matching as nearest neighbor caliper matching (see Appendix Tables A2a‐c and Appendix Tables A3a‐c). After removing all unsuccessful matched sets, there were still sets yielding a 95% confidence interval that overlapped 1, while other sets did not, both with caliper matching as NN caliper matching.

### 3.3 Real life dataset

**FIGURE 2:**
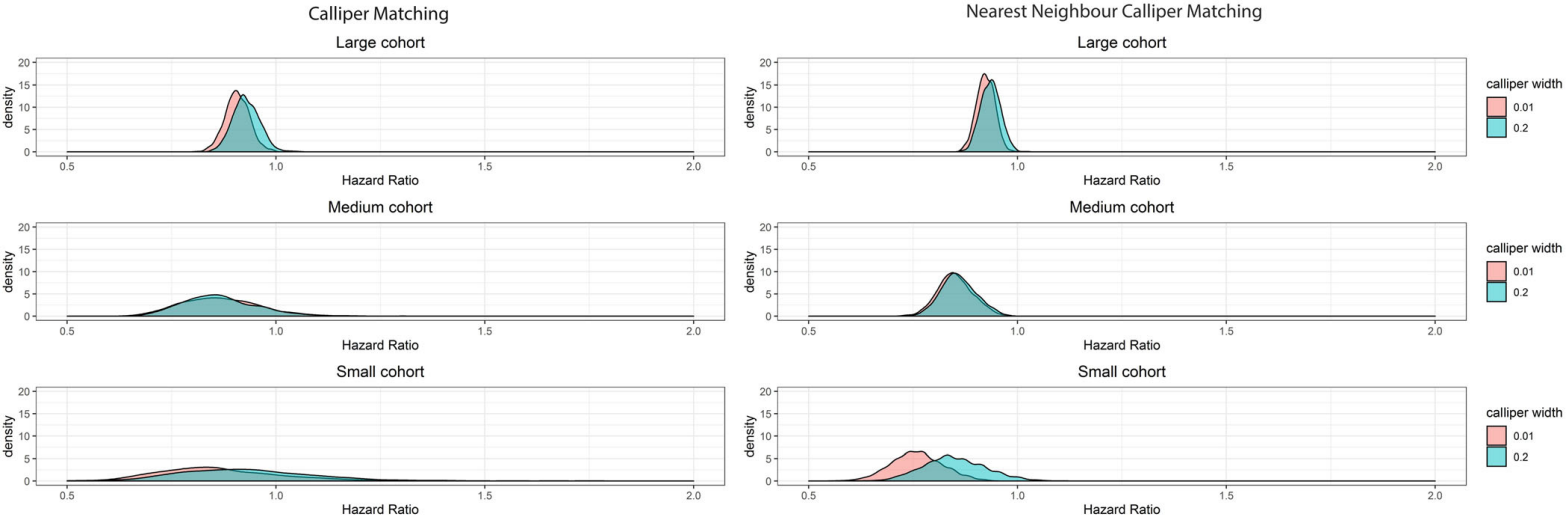
Density plots of the distribution of the hazard ratio of the 1000 matched sets from the three Stockholm atrial fibrillation cohorts after caliper matching and after nearest neighbor caliper matching [Colour figure can be viewed at wileyonlinelibrary.com]

In line with the simulations, the largest variation was visible in the smallest cohort (n = 1594) after caliper matching with a median HR of 0.94 (IQR: 0.82‐1.06), ranging from 0.52 (CI: 0.27‐1.01) to 2.43 (CI: 1.01‐5.86). The variation was smaller in the large cohort and after NN caliper matching (see Figure 2 and Table 5). Again, the 95% confidence was nonconsistently overlapping 1 after repeating the matches.

**TABLE 5:** Results real life observational study

	Median HR	Interquartile range HR	Full range HR	% sign low risk	% sign high risk
Large cohort (n = 18 203)					
Caliper matching	0.91	0.04 (0.89‐0.93)	0.19 (0.81‐0.99)	4.7%	0.0%
Nearest neighbor caliper matching	0.92	0.03 (0.91‐0.94)	0.14 (0.86‐1.00)	0.0%	0.0%
Medium cohort (n = 5696)					
Caliper matching	0.86	0.13 (0.80‐0.93)	0.67 (0.65‐1.32)	2.7%	0.0%
Nearest neighbor caliper matching	0.85	0.06 (0.82‐0.88)	0.25 (0.72‐0.97)	0.1%	0.0%
Small cohort (n = 1594)					
Caliper matching	0.94	0.24 (0.82‐1.06)	1.91 (0.52‐2.43)	0.0%	0.1%
Nearest neighbor caliper matching	0.76	0.09 (0.71‐0.81)	0.35 (0.57‐0.92)	0.0%	0.0%

*Note:* Results from the 1000 matched sets in the three sizes of the Stockholm AF cohort. HR = hazard ratio; % sign low/high risk = percentage of the 1000 matched sets with a significantly increased or decreased risk.

## 4 DISCUSSION

We used simulations and an empirical example to illustrate that there can be large variation in point estimates when repeatedly applying the greedy caliper PS matching algorithm, in which patients are randomly ordered, on the same cohort. This variability was, to a lesser extent, also visible when applying greedy NN caliper PS matching. With increasing sample sizes, the variation decreased, but whether a value of 1 was within the estimated confidence intervals after matching was inconsistent after replication. In simulated cohorts with low outcome prevalence, the variability was largest, while in these situations, propensity score methods (and thus matching) are frequently used. Using a real‐life dataset comparing NOACs to VKAs and the risk for stroke, we confirmed these findings.

From our literature search, we found that the NN matching algorithm was the most commonly used matching method, followed by caliper matching. We found that the MatchIt package was the most frequently used software package for matching (and the only package mentioned when using R), and in this package it is not possible to perform NN caliper matching, but only caliper matching without NN or NN matching without caliper. Interestingly, nine papers specifically mentioned they used the MatchIt package for NN caliper matching, and it could be those papers actually performed caliper matching without NN, with the risk of high variability. As the statistical package is not mentioned in most articles (n = 79), it is not possible to determine how the matching procedure took place. We recommend better reporting of matching procedures used, including which statistical software, as this can ultimately affect the results of a study, and is necessary for study replication.

In addition, we found that approximately 50% of the studies were conducted in a cohort with a sample size of 2500 or less. In our simulation study, we showed that in these sample sizes the treatment effects are largely influenced by the selected random seed, which in practice is not often specified or reported. In addition, whether the 95% CI overlapped 1 was inconsistent with different random seeds. However, this can also be a result of too little power in the limited sample sizes. But still, results of studies using caliper PS matching in datasets with these sample sizes should be interpreted with caution, as the choice of starting seed for the matching algorithm could be manipulated to yield a significant test statistic.

One way researchers often show whether matching was successful or not, is by showing the SMD for all covariates^11^. It is common practice to consider matching successful when the SMD for all covariates is below 0.1^12^. In our simulation study, we showed that the SMDs are also dependent on the random seed that is used, in particular in datasets with small sample sizes. Removing matched sets with unsuccessful matching only slightly decreased the variation of the point estimates. Therefore, repeating the matching until all SMDs are below 0.1 will not solve the issue of variability.

With caliper matching, we found that the median OR of the 1000 repetitions was close to the simulated parameter, while in NN caliper matching in some instances the median OR was not as expected, indicating this approach might introduce some bias. Potentially, a future direction could be to apply repeated caliper PS matching and use the mean or median for the point estimate, as this is independent of the random ordering. Approaches have been made in using bagged one‐to‐one matching, which overcomes the variability introduced by the matching through bagging (ie, use bootstrapping to resample a cohort and propensity score match and analyze all resamples)^14,15^, but it remains unknown how this approach would compare to repeated caliper PS matching.

To avoid the proposed problem, we suggest that researchers stop using greedy caliper matching with random ordering. In addition, the use of NN caliper matching should be reconsidered, as there are alternative propensity score matching methods that are not affected by random variability, such as optimal matching^13^. The NN matching procedure can also yield findings independent of random ordering. For example, if treated patients are not ordered at random prior to matching, if the algorithm is performed without calipers and with replacement, or if the best match is selected at first. However, it is not within the scope of the current research to make statements on which approach is preferred.

In conclusion, replication of greedy caliper PS matching in the same cohort can yield highly variable estimates of the treatment‐outcome association, already in moderately sized cohorts of 2500 patients. To avoid the problem of random variability in point estimates, researchers should refrain from using versions of greedy matching that are dependent on random ordering and/or random within caliper matching. If a greedy matching algorithm is used, nearest neighbor within caliper matching combined with nonrandom ordering (eg, best first, ascending, descending) would be preferred.

## CONFLICT OF INTEREST

J.K. reports personal fees from Boehringer Ingelheim, outside of the submitted work. S.S. is participating in investigator‐initiated grants to the Brigham and Women's Hospital from Bayer, Vertex, and Boehringer Ingelheim, outside of the submitted work. He is a consultant to Aetion Inc., a software manufacturer of which he owns equity. His interests were declared, reviewed, and approved by the Brigham and Women's Hospital and Partners HealthCare System in accordance with their institutional compliance policies. S.B., R.W., A.T., T.F., R.P., and O.K. have nothing to disclose. ICMJE Conflict of Interest forms are available with the authors on request.

## ETHICS STATEMENT

The study was approved by the Regional Ethical Review Board in Stockholm (EPN 2015/579–31/2).

## Data Availability

Data from the Stockholm Healthcare Database is not allowed to be transferred. The code for the simulation study and for the modifications in the MatchIt package is available with the authors on request.

## Appendix A.

### A..1 eResults 1: Caliper width of 0.01 SD_ps_

**TABLE A1a:** Results of repeated calliper and nearest neighbor calliper propensity score matching in cohorts with a simulated odds ratio of 0.75

		Median OR	Interquartile range OR	Full range OR	% Sign low risk	% Sign high risk	Mean n matches	% Unsuccessful matches
N = 10.000								
Caliper Matching	T 50%, O 50%	0.77	(0.76‐0.78)	(0.72‐0.82)	100.0%	0.0%	7069	0.0%
	T 20%, O 50%	0.77	(0.75‐0.78)	(0.67‐0.85)	100.0%	0.0%	3752	0.0%
	T 50%, O 10%	0.78	(0.76‐0.80)	(0.69‐0.90)	99.5%	0.0%	6836	0.0%
	T 20%, O 10%	0.79	(0.76‐0.83)	(0.59‐1.01)	43.8%	0.0%	3752	0.0%
Nearest Neighbor Caliper Matching	T 50%, O 50%	0.76	(0.75‐0.77)	(0.72‐0.79)	100.0%	0.0%	7041	0.0%
	T 20%, O 50%	0.73	(0.72‐0.73)	(0.71‐0.75)	100.0%	0.0%	3747	0.0%
	T 50%, O 10%	0.77	(0.76‐0.78)	(0.70‐0.84)	100.0%	0.0%	6811	0.0%
	T 20%, O 10%	0.84	(0.84‐0.85)	(0.81‐0.88)	0.0%	0.0%	3747	0.0%
N = 2500								
Caliper Matching	T 50%, O 50%	0.77	(0.74‐0.79)	(0.68‐0.86)	96.4%	0.0%	1722	0.0%
	T 20%, O 50%	0.82	(0.78‐0.86)	(0.62‐1.04)	21.5%	0.0%	948	8.7%
	T 50%, O 10%	0.73	(0.70‐0.77)	(0.58‐0.88)	30.2%	0.0%	1722	0.0%
	T 20%, O 10%	0.77	(0.70‐0.83)	(0.51‐1.14)	5.4%	0.0%	931	3.3%
Nearest Neighbor Caliper Matching	T 50%, O 50%	0.76	(0.75‐0.78)	(0.70‐0.85)	99.5%	0.0%	1701	0.0%
	T 20%, O 50%	0.78	(0.76‐0.79)	(0.72‐0.83)	37.9%	0.0%	942	0.0%
	T 50%, O 10%	0.84	(0.82‐0.87)	(0.73‐0.97)	0.0%	0.0%	1701	0.0%
	T 20%, O 10%	0.82	(0.79‐0.84)	(0.74‐0.94)	0.0%	0.0%	930	0.0%
N = 500								
Caliper Matching	T 50%, O 50%	0.76	(0.71‐0.80)	(0.58‐1.00)	0.9%	0.0%	252	84.9%
	T 20%, O 50%	0.50	(0.46‐0.56)	(0.27‐0.89)	55.6%	0.0%	152	87.0%
	T 50%, O 10%	0.80	(0.71‐0.91)	(0.47‐1.22)	0.0%	0.0%	244	100.0%
	T 20%, O 10%	0.75	(0.67‐0.86)	(0.38‐1.50)	0.1%	0.0%	143	100.0%
Nearest Neighbor Caliper Matching	T 50%, O 50%	0.73	(0.69‐0.76)	(0.58‐0.97)	0.2%	0.0%	249	77.4%
	T 20%, O 50%	0.46	(0.43‐0.48)	(0.38‐0.52)	100.0%	0.0%	152	100.0%
	T 50%, O 10%	0.92	(0.83‐0.92)	(0.75‐1.00)	0.0%	0.0%	242	100.0%
	T 20%, O 10%	0.83	(0.71‐0.83)	(0.71‐0.83)	0.0%	0.0%	142	100.0%

*Note:* The calliper was set at 0.01 of the SD of the propensity score. The % sign low and sign high risk state in how many of the successfully matched cohorts the treatment‐outcome associations was statistically different from 1. The % unsuccessful matches is in how many instances there was at least one of the SMDs above 0.1.

Abbreviation: OR, odds ratio.

**TABLE A1b:** Results of repeated calliper and nearest neighbor calliper propensity score matching in cohorts with a simulated odds ratio of 1.0

		Median OR	Interquartile range OR	Full range OR	% sign low risk	% sign high risk	Mean n matches	% unsuccessful matches
N = 10.000								
Caliper matching	T 50%, O 50%	1.02	(1.00‐1.03)	(0.95‐1.09)	0.0%	0.0%	7069	0.0%
	T 20%, O 50%	1.01	(0.99‐1.03)	(0.90‐1.13)	0.0%	0.0%	3752	0.0%
	T 50%, O 10%	1.01	(0.98‐1.03)	(0.88‐1.17)	0.0%	0.1%	6836	0.0%
	T 20%, O 10%	1.06	(1.01‐1.11)	(0.81‐1.33)	0.0%	1.0%	3752	0.0%
Nearest neighbor caliper matching	T 50%, O 50%	1.01	(1.00‐1.02)	(0.97‐1.06)	0.0%	0.0%	7041	0.0%
	T 20%, O 50%	0.96	(0.96‐0.97)	(0.93‐1.00)	0.0%	0.0%	3747	0.0%
	T 50%, O 10%	1.00	(0.98‐1.01)	(0.93‐1.07)	0.0%	0.0%	6811	0.0%
	T 20%, O 10%	1.13	(1.12‐1.14)	(1.08‐1.18)	0.0%	0.0%	3747	0.0%
N = 2500								
Caliper matching	T 50%, O 50%	1.00	(0.97‐1.03)	(0.86‐1.15)	0.0%	0.0%	1621	0.0%
	T 20%, O 50%	1.08	(1.03‐1.14)	(0.87‐1.38)	0.0%	0.5%	871	12.8%
	T 50%, O 10%	1.05	(1.01‐1.10)	(0.83‐1.34)	0.0%	0.0%	1672	0.1%
	T 20%, O 10%	1.12	(1.03‐1.26)	(0.71‐1.89)	0.0%	0.5%	838	7.9%
Nearest neighbor caliper matching	T 50%, O 50%	1.02	(0.99‐1.04)	(0.93‐1.12)	0.0%	0.0%	1605	0.0%
	T 20%, O 50%	1.01	(1.00‐1.02)	(0.95‐1.08)	0.0%	0.0%	869	0.3%
	T 50%, O 10%	1.04	(1.00‐1.07)	(0.88‐1.21)	0.0%	0.0%	1651	0.0%
	T 20%, O 10%	1.33	(1.30‐1.35)	(1.25‐1.38)	0.0%	0.0%	840	0.0%
N = 500								
Caliper matching	T 50%, O 50%	0.93	(0.87‐0.97)	(0.65‐1.22)	0.0%	0.0%	245	99.0%
	T 20%, O 50%	0.78	(0.70‐0.85)	(0.50‐1.29)	0.1%	0.0%	135	99.3%
	T 50%, O 10%	1.00	(0.90‐1.11)	(0.50‐1.83)	0.0%	0.0%	239	57.3%
	T 20%, O 10%	1.25	(1.00‐1.25)	(0.56‐2.50)	0.0%	0.0%	135	99.3%
Nearest neighbor caliper matching	T 50%, O 50%	0.93	(0.88‐1.00)	(0.68‐1.26)	0.0%	0.0%	241	100.0%
	T 20%, O 50%	0.89	(0.85‐0.90)	(0.81‐0.95)	0.0%	0.0%	136	100.0%
	T 50%, O 10%	1.00	(1.00‐1.10)	(0.73‐1.40)	0.0%	0.0%	240	54.7%
	T 20%, O 10%	1.25	(1.25‐1.25)	(1.25‐1.25)	0.0%	0.0%	136	100.0%

*Note:* The calliper was set at 0.01 of the SD of the propensity score. The % sign low and sign high risk state in how many of the successfully matched cohorts the treatment‐outcome associations was statistically different from 1. The % unsuccessful matches is in how many instances there was at least one of the SMDs above 0.1.

Abbreviation: OR, odds ratio.

**TABLE A1c:** Results of repeated calliper and nearest neighbor calliper propensity score matching in cohorts with a simulated odds ratio of 1.5

		Median OR	Interquartile range OR	Full range OR	% sign low risk	% sign high risk	Mean n matches	% unsuccessful matches
N = 10.000								
Caliper Matching	T 50%, O 50%	1.46	(1.43‐1.48)	(1.36‐1.58)	0.0%	100.0%	7069	0.0%
	T 20%, O 50%	1.47	(1.44‐1.51)	(1.30‐1.63)	0.0%	100.0%	3752	0.0%
	T 50%, O 10%	1.44	(1.41‐1.47)	(1.27‐1.62)	0.0%	100.0%	6836	0.0%
	T 20%, O 10%	1.54	(1.46‐1.60)	(1.24‐1.94)	0.0%	100.0%	3700	0.0%
Nearest Neighbor Caliper Matching	T 50%, O 50%	1.45	(1.43‐1.46)	(1.37‐1.51)	0.0%	100.0%	7041	0.0%
	T 20%, O 50%	1.40	(1.39‐1.41)	(1.35‐1.44)	0.0%	100.0%	3747	0.0%
	T 50%, O 10%	1.42	(1.40‐1.45)	(1.33‐1.51)	0.0%	100.0%	6811	0.0%
	T 20%, O 10%	1.54	(1.53‐1.56)	(1.48‐1.60)	0.0%	100.0%	3689	0.0%
N = 2500								
Caliper matching	T 50%, O 50%	1.45	(1.40‐1.49)	(1.27‐1.74)	0.0%	100.0%	1672	0.1%
	T 20%, O 50%	1.39	(1.33‐1.47)	(1.12‐1.75)	0.0%	83.0%	931	3.3%
	T 50%, O 10%	1.41	(1.35‐1.48)	(1.11‐1.73)	0.0%	77.2%	1621	0.0%
	T 20%, O 10%	1.49	(1.36‐1.61)	(0.98‐2.24)	0.0%	41.0%	931	3.3%
Nearest neighbor caliper matching	T 50%, O 50%	1.49	(1.45‐1.52)	(1.35‐1.63)	0.0%	100.0%	1651	0.0%
	T 20%, O 50%	1.45	(1.43‐1.47)	(1.34‐1.57)	0.0%	100.0%	930	0.0%
	T 50%, O 10%	1.48	(1.44‐1.52)	(1.30‐1.65)	0.0%	99.1%	1605	0.0%
	T 20%, O 10%	1.57	(1.53‐1.62)	(1.42‐1.81)	0.0%	80.9%	930	0.0%
N = 500								
Caliper matching	T 50%, O 50%	1.48	(1.38‐1.60)	(1.04‐2.00)	0.0%	9.0%	240	70.4%
	T 20%, O 50%	1.37	(1.26‐1.50)	(0.89‐2.09)	0.0%	0.5%	134	98.3%
	T 50%, O 10%	1.56	(1.40‐1.78)	(0.91‐3.40)	0.0%	1.6%	239	57.3%
	T 20%, O 10%	1.14	(1.00‐1.33)	(0.62‐3.00)	0.0%	0.0%	126	98.4%
Nearest neighbor caliper matching	T 50%, O 50%	1.58	(1.50‐1.70)	(1.21‐2.13)	0.0%	13.6%	239	82.2%
	T 20%, O 50%	1.24	(1.18‐1.27)	(1.06‐1.43)	0.0%	0.0%	132	100.0%
	T 50%, O 10%	1.60	(1.50‐1.70)	(1.09‐2.11)	0.0%	0.0%	240	54.7%
	T 20%, O 10%	1.14	(1.14‐1.14)	(1.14‐1.14)	0.0%	0.0%	125	100.0%

*Note:* The calliper was set at 0.01 of the SD of the propensity score. The % sign low and sign high risk state in how many of the successfully matched cohorts the treatment‐outcome associations was statistically different from 1. The % unsuccessful matches is in how many instances there was at least one of the SMDs above 0.1.

Abbreviation: OR, odds ratio.

### A..2 eResults 2: Successful matches with caliper width of 0.2 SD_ps_

**TABLE A2a:** Results of repeated calliper and nearest neighbor calliper propensity score matching in cohorts with a simulated odds ratio of 0.75 where the SMD for all covariates was ≤0.1 after matching

		Median OR	Interquartile range OR	Full range OR	% sign low risk	% sign high risk
N = 10.000						
Caliper Matching	T 50%, O 50%	0.75	(0.74‐0.76)	(0.69‐0.81)	100.0%	0.0%
	T 20%, O 50%	0.78	(0.76‐0.80)	(0.70‐0.89)	99.9%	0.0%
	T 50%, O 10%	0.76	(0.74‐0.78)	(0.67‐0.84)	100.0%	0.0%
	T 20%, O 10%	0.79	(0.76‐0.83)	(0.66‐1.03)	45.4%	0.0%
Nearest Neighbor Caliper Matching	T 50%, O 50%	0.75	(0.74‐0.76)	(0.71‐0.79)	100.0%	0.0%
	T 20%, O 50%	0.74	(0.73‐0.74)	(0.72‐0.76)	100.0%	0.0%
	T 50%, O 10%	0.75	(0.74‐0.76)	(0.70‐0.80)	100.0%	0.0%
	T 20%, O 10%	0.86	(0.85‐0.86)	(0.83‐0.89)	0.0%	0.0%
N = 2500						
Caliper Matching	T 50%, O 50%	0.78	(0.76‐0.79)	(0.69‐0.86)	97.6%	0.0%
	T 20%, O 50%	0.78	(0.74‐0.82)	(0.61‐1.02)	48.1%	0.0%
	T 50%, O 10%	0.72	(0.69‐0.75)	(0.56‐0.90)	48.2%	0.0%
	T 20%, O 10%	0.74	(0.69‐0.81)	(0.55‐1.13)	10.4%	0.0%
Nearest Neighbor Caliper Matching	T 50%, O 50%	0.78	(0.76‐0.79)	(0.70‐0.84)	99.6%	0.0%
	T 20%, O 50%	0.76	(0.75‐0.77)	(0.72‐0.82)	91.6%	0.0%
	T 50%, O 10%	0.77	(0.75‐0.79)	(0.67‐0.88)	3.0%	0.0%
	T 20%, O 10%	0.78	(0.76‐0.80)	(0.69‐0.88)	0.0%	0.0%
N = 500						
Caliper Matching	T 50%, O 50%	0.73	(0.68‐0.78)	(0.51‐1.00)	6.9%	0.0%
	T 20%, O 50%	0.73	(0.64‐0.80)	(0.49‐1.13)	2.0%	0.0%
	T 50%, O 10%	0.68	(0.61‐0.75)	(0.33‐1.07)	0.6%	0.0%
	T 20%, O 10%	0.78	(0.67‐1.00)	(0.43‐2.33)	0.0%	0.0%
Nearest Neighbor Caliper Matching	T 50%, O 50%	0.69	(0.65‐0.74)	(0.53‐0.91)	10.1%	0.0%
	T 20%, O 50%	0.66	(0.63‐0.67)	(0.58‐0.71)	0.1%	0.0%
	T 50%, O 10%	0.86	(0.80‐0.93)	(0.57‐1.08)	0.0%	0.0%
	T 20%, O 10%	0.71	(0.67‐0.71)	(0.63‐0.75)	0.0%	0.0%

*Note:* The calliper was set at 0.2 of the SD of the propensity score. The % sign low and sign high risk state in how many of the successfully matched cohorts the treatment‐outcome associations was statistically different from 1.

Abbreviation: OR, odds ratio.

**TABLE A2b:** Results of repeated calliper and nearest neighbor calliper propensity score matching in cohorts with a simulated odds ratio of 1.0 where the SMD for all covariates was ≤0.1 after matching

		Median OR	Interquartile range OR	Full range OR	% sign low risk	% sign high risk
N = 10.000						
Caliper Matching	T 50%, O 50%	0.99	(0.98‐1.01)	(0.91‐1.04)	0.0%	0.0%
	T 20%, O 50%	1.02	(1.00‐1.05)	(0.92‐1.17)	0.0%	0.2%
	T 50%, O 10%	0.98	(0.96‐1.00)	(0.90‐1.07)	0.0%	0.0%
	T 20%, O 10%	1.05	(1.01‐1.10)	(0.87‐1.37)	0.0%	0.5%
Nearest Neighbor Caliper Matching	T 50%, O 50%	1.00	(0.99‐1.01)	(0.96‐1.05)	0.0%	0.0%
	T 20%, O 50%	0.97	(0.97‐0.98)	(0.95‐1.00)	0.0%	0.0%
	T 50%, O 10%	0.97	(0.96‐0.99)	(0.91‐1.04)	0.0%	0.0%
	T 20%, O 10%	1.14	(1.13‐1.15)	(1.09‐1.18)	0.0%	0.0%
N = 2500						
Caliper Matching	T 50%, O 50%	0.94	(0.92‐0.97)	(0.79‐1.10)	0.2%	0.0%
	T 20%, O 50%	1.02	(0.96‐1.07)	(0.77‐1.30)	0.0%	0.0%
	T 50%, O 10%	1.01	(0.98‐1.05)	(0.85‐1.17)	0.0%	0.0%
	T 20%, O 10%	1.00	(0.89‐1.10)	(0.65‐1.72)	0.0%	0.0%
Nearest Neighbor Caliper Matching	T 50%, O 50%	0.98	(0.96‐1.00)	(0.90‐1.08)	0.0%	0.0%
	T 20%, O 50%	0.97	(0.96‐0.98)	(0.91‐1.03)	0.0%	0.0%
	T 50%, O 10%	0.95	(0.92‐0.98)	(0.82‐1.07)	0.0%	0.0%
	T 20%, O 10%	1.24	(1.21‐1.25)	(1.13‐1.36)	0.0%	0.0%
N = 500						
Caliper Matching	T 50%, O 50%	1.02	(0.97‐1.09)	(0.73‐1.35)	0.0%	0.0%
	T 20%, O 50%	0.96	(0.86‐1.09)	(0.61‐1.35)	0.0%	0.0%
	T 50%, O 10%	1.00	(0.88‐1.13)	(0.53‐2.57)	0.0%	0.1%
	T 20%, O 10%	1.00	(0.83‐1.20)	(0.40‐3.50)	0.0%	0.0%
Nearest Neighbor Caliper Matching	T 50%, O 50%	1.08	(1.03‐1.13)	(0.89‐1.34)	0.0%	0.0%
	T 20%, O 50%	1.08	(1.04‐1.12)	(0.92‐1.23)	0.0%	0.0%
	T 50%, O 10%	1.00	(0.93‐1.07)	(0.67‐1.43)	0.0%	0.0%
	T 20%, O 10%	1.00	(1.00‐1.00)	(0.83‐1.00)	0.0%	0.0%

*Note:* The calliper was set at 0.2 of the SD of the propensity score. The % sign low and sign high risk state in how many of the successfully matched cohorts the treatment‐outcome associations was statistically different from 1.

Abbreviation: OR, odds ratio.

**TABLE A2c:** Results of repeated calliper and nearest neighbor calliper propensity score matching in cohorts with a simulated odds ratio of 1.5 where the SMD for all covariates was ≤0.1 after matching

		Median OR	Interquartile range OR	Full range OR	% sign low risk	% sign high risk
N = 10.000						
Caliper Matching	T 50%, O 50%	1.42	(1.40‐1.44)	(1.33‐1.51)	0.0%	100.0%
	T 20%, O 50%	1.50	(1.46‐1.54)	(1.34‐1.69)	0.0%	100.0%
	T 50%, O 10%	1.41	(1.38‐1.44)	(1.29‐1.55)	0.0%	100.0%
	T 20%, O 10%	1.49	(1.43‐1.57)	(1.21‐1.95)	0.0%	99.8%
Nearest Neighbor Caliper Matching	T 50%, O 50%	1.43	(1.41‐1.44)	(1.35‐1.50)	0.0%	100.0%
	T 20%, O 50%	1.42	(1.41‐1.42)	(1.38‐1.46)	0.0%	100.0%
	T 50%, O 10%	1.39	(1.37‐1.41)	(1.29‐1.48)	0.0%	100.0%
	T 20%, O 10%	1.51	(1.50‐1.53)	(1.46‐1.57)	0.0%	100.0%
N = 2500						
Caliper Matching	T 50%, O 50%	1.43	(1.40‐1.47)	(1.26‐1.62)	0.0%	100.0%
	T 20%, O 50%	1.40	(1.34‐1.47)	(1.13‐1.79)	0.0%	86.1%
	T 50%, O 10%	1.45	(1.39‐1.51)	(1.15‐1.73)	0.0%	92.0%
	T 20%, O 10%	1.44	(1.33‐1.56)	(1.04‐2.29)	0.0%	36.5%
Nearest Neighbor Caliper Matching	T 50%, O 50%	1.48	(1.46‐1.51)	(1.35‐1.61)	0.0%	100.0%
	T 20%, O 50%	1.44	(1.42‐1.46)	(1.34‐1.55)	0.0%	100.0%
	T 50%, O 10%	1.49	(1.45‐1.53)	(1.29‐1.69)	0.0%	99.8%
	T 20%, O 10%	1.47	(1.44‐1.50)	(1.33‐1.64)	0.0%	38.2%
N = 500						
Caliper Matching	T 50%, O 50%	1.41	(1.31‐1.50)	(1.02‐2.04)	0.0%	9.8%
	T 20%, O 50%	1.29	(1.18‐1.44)	(0.91‐1.85)	0.0%	0.1%
	T 50%, O 10%	1.50	(1.35‐1.69)	(0.90‐3.86)	0.0%	3.3%
	T 20%, O 10%	1.25	(1.11‐1.67)	(0.64‐3.00)	0.0%	0.0%
Nearest Neighbor Caliper Matching	T 50%, O 50%	1.37	(1.29‐1.45)	(1.03‐1.85)	0.0%	2.8%
	T 20%, O 50%	1.04	(1.00‐1.04)	(0.93‐1.12)	0.0%	0.0%
	T 50%, O 10%	1.50	(1.40‐1.60)	(1.07‐2.00)	0.0%	0.2%
	T 20%, O 10%	#N/A	#N/A	#N/A	0.0%	0.0%

*Note:* The calliper was set at 0.2 of the SD of the propensity score. The % sign low and sign high risk state in how many of the successfully matched cohorts the treatment‐outcome associations was statistically different from 1.

Abbreviation: OR, odds ratio.

### A..3 eResults 3: Successful matches with caliper width of 0.01 SD_ps_

**TABLE A3a:** Results of repeated calliper and nearest neighbor calliper propensity score matching in cohorts with a simulated odds ratio of 0.75 where the SMD for all covariates was ≤0.1 after matching

		Median OR	Interquartile range OR	Full range OR	% sign low risk	% sign high risk
N = 10.000						
Caliper Matching	T 50%, O 50%	0.77	(0.76‐0.78)	(0.72‐0.82)	100.0%	0.0%
	T 20%, O 50%	0.77	(0.75‐0.78)	(0.67‐0.85)	100.0%	0.0%
	T 50%, O 10%	0.78	(0.76‐0.80)	(0.69‐0.90)	99.5%	0.0%
	T 20%, O 10%	0.79	(0.76‐0.83)	(0.59‐1.01)	43.8%	0.0%
Nearest Neighbor Caliper Matching	T 50%, O 50%	0.76	(0.75‐0.77)	(0.72‐0.79)	100.0%	0.0%
	T 20%, O 50%	0.73	(0.72‐0.73)	(0.71‐0.75)	100.0%	0.0%
	T 50%, O 10%	0.77	(0.76‐0.78)	(0.70‐0.84)	100.0%	0.0%
	T 20%, O 10%	0.84	(0.84‐0.85)	(0.81‐0.88)	0.0%	0.0%
N = 2500						
Caliper Matching	T 50%, O 50%	0.77	(0.74‐0.79)	(0.68‐0.86)	96.4%	0.0%
	T 20%, O 50%	0.82	(0.78‐0.85)	(0.62‐1.04)	19.9%	0.0%
	T 50%, O 10%	0.73	(0.70‐0.77)	(0.58‐0.88)	30.2%	0.0%
	T 20%, O 10%	0.77	(0.70‐0.83)	(0.51‐1.14)	5.3%	0.0%
Nearest Neighbor Caliper Matching	T 50%, O 50%	0.76	(0.75‐0.78)	(0.70‐0.85)	99.5%	0.0%
	T 20%, O 50%	0.78	(0.76‐0.79)	(0.72‐0.83)	37.9%	0.0%
	T 50%, O 10%	0.84	(0.82‐0.87)	(0.73‐0.97)	0.0%	0.0%
	T 20%, O 10%	0.82	(0.79‐0.84)	(0.74‐0.94)	0.0%	0.0%
N = 500						
Caliper Matching	T 50%, O 50%	0.76	(0.72‐0.81)	(0.58‐0.94)	0.1%	0.0%
	T 20%, O 50%	0.50	(0.46‐0.57)	(0.31‐0.70)	7.0%	0.0%
	T 50%, O 10%	#N/A	#N/A	#N/A	0.0%	0.0%
	T 20%, O 10%	#N/A	#N/A	#N/A	0.0%	0.0%
Nearest Neighbor Caliper Matching	T 50%, O 50%	0.73	(0.69‐0.76)	(0.60‐0.89)	0.1%	0.0%
	T 20%, O 50%	#N/A	#N/A	#N/A	0.0%	0.0%
	T 50%, O 10%	#N/A	#N/A	#N/A	0.0%	0.0%
	T 20%, O 10%	#N/A	#N/A	#N/A	0.0%	0.0%

*Note:* The calliper was set at 0.01 of the standard deviation of the propensity score. The % sign low and sign high risk state in how many of the successfully matched cohorts the treatment‐outcome associations was statistically different from 1.

Abbreviation: OR, odds ratio.

**TABLE A3b:** Results of repeated calliper and nearest neighbor calliper propensity score matching in cohorts with a simulated odds ratio of 1.0 where the SMD for all covariates was ≤0.1 after matching

		Median OR	Interquartile range OR	Full range OR	% sign low risk	% sign high risk
N = 10.000						
Caliper Matching	T 50%, O 50%	1.02	(1.00‐1.03)	(0.95‐1.09)	0.0%	0.0%
	T 20%, O 50%	1.01	(0.99‐1.03)	(0.90‐1.13)	0.0%	0.0%
	T 50%, O 10%	1.01	(0.98‐1.03)	(0.88‐1.17)	0.0%	0.1%
	T 20%, O 10%	1.06	(1.01‐1.11)	(0.81‐1.33)	0.0%	1.0%
Nearest Neighbor Caliper Matching	T 50%, O 50%	1.01	(1.00‐1.02)	(0.97‐1.06)	0.0%	0.0%
	T 20%, O 50%	0.96	(0.96‐0.97)	(0.93‐1.00)	0.0%	0.0%
	T 50%, O 10%	1.00	(0.98‐1.01)	(0.93‐1.07)	0.0%	0.0%
	T 20%, O 10%	1.13	(1.12‐1.14)	(1.08‐1.18)	0.0%	0.0%
N = 2500						
Caliper Matching	T 50%, O 50%	1.00	(0.97‐1.03)	(0.86‐1.15)	0.0%	0.0%
	T 20%, O 50%	1.08	(1.02‐1.14)	(0.87‐1.38)	0.0%	0.5%
	T 50%, O 10%	1.05	(1.01‐1.10)	(0.83‐1.34)	0.0%	0.0%
	T 20%, O 10%	1.11	(1.03‐1.24)	(0.71‐1.89)	0.0%	0.5%
Nearest Neighbor Caliper Matching	T 50%, O 50%	1.02	(0.99‐1.04)	(0.93‐1.12)	0.0%	0.0%
	T 20%, O 50%	1.01	(1.00‐1.02)	(0.95‐1.08)	0.0%	0.0%
	T 50%, O 10%	1.04	(1.00‐1.07)	(0.88‐1.21)	0.0%	0.0%
	T 20%, O 10%	1.33	(1.30‐1.35)	(1.25‐1.38)	0.0%	0.0%
N = 500						
Caliper Matching	T 50%, O 50%	0.90	(0.85‐0.93)	(0.71‐1.10)	0.0%	0.0%
	T 20%, O 50%	0.78	(0.74‐0.80)	(0.73‐0.85)	0.0%	0.0%
	T 50%, O 10%	1.00	(0.90‐1.13)	(0.54‐1.83)	0.0%	0.0%
	T 20%, O 10%	1.25	(1.13‐1.88)	(0.83‐2.50)	0.0%	0.0%
Nearest Neighbor Caliper Matching	T 50%, O 50%	#N/A	#N/A	#N/A	0.0%	0.0%
	T 20%, O 50%	#N/A	#N/A	#N/A	0.0%	0.0%
	T 50%, O 10%	1.00	(1.00‐1.10)	(0.73‐1.40)	0.0%	0.0%
	T 20%, O 10%	#N/A	#N/A	#N/A	0.0%	0.0%

*Note:* The calliper was set at 0.01 of the standard deviation of the propensity score. The % sign low and sign high risk state in how many of the successfully matched cohorts the treatment‐outcome associations was statistically different from 1.

Abbreviation: OR, odds ratio.

**TABLE A3c:** Results of repeated calliper and nearest neighbor calliper propensity score matching in cohorts with a simulated odds ratio of 1.5 where the SMD for all covariates was ≤0.1 after matching

		Median OR	Interquartile range OR	Full range OR	% sign low risk	% sign high risk
N = 10.000						
Caliper Matching	T 50%, O 50%	1.46	(1.43‐1.48)	(1.36‐1.58)	0.0%	100.0%
	T 20%, O 50%	1.47	(1.44‐1.51)	(1.30‐1.63)	0.0%	100.0%
	T 50%, O 10%	1.44	(1.41‐1.47)	(1.27‐1.62)	0.0%	100.0%
	T 20%, O 10%	1.54	(1.46‐1.60)	(1.24‐1.94)	0.0%	100.0%
Nearest Neighbor Caliper Matching	T 50%, O 50%	1.45	(1.43‐1.46)	(1.37‐1.51)	0.0%	100.0%
	T 20%, O 50%	1.40	(1.39‐1.41)	(1.35‐1.44)	0.0%	100.0%
	T 50%, O 10%	1.42	(1.40‐1.45)	(1.33‐1.51)	0.0%	100.0%
	T 20%, O 10%	1.54	(1.53‐1.56)	(1.48‐1.60)	0.0%	100.0%
N = 2500						
Caliper Matching	T 50%, O 50%	1.45	(1.40‐1.49)	(1.27‐1.72)	0.0%	99.9%
	T 20%, O 50%	1.39	(1.32‐1.47)	(1.12‐1.75)	0.0%	79.9%
	T 50%, O 10%	1.41	(1.35‐1.48)	(1.11‐1.73)	0.0%	77.2%
	T 20%, O 10%	1.48	(1.36‐1.62)	(0.98‐2.24)	0.0%	39.4%
Nearest Neighbor Caliper Matching	T 50%, O 50%	1.49	(1.45‐1.52)	(1.35‐1.63)	0.0%	100.0%
	T 20%, O 50%	1.45	(1.43‐1.47)	(1.34‐1.57)	0.0%	100.0%
	T 50%, O 10%	1.48	(1.44‐1.52)	(1.30‐1.65)	0.0%	99.1%
	T 20%, O 10%	1.57	(1.53‐1.62)	(1.42‐1.81)	0.0%	80.9%
N = 500						
Caliper Matching	T 50%, O 50%	1.46	(1.36‐1.57)	(1.12‐2.00)	0.0%	2.1%
	T 20%, O 50%	1.37	(1.24‐1.41)	(0.95‐1.60)	0.0%	0.0%
	T 50%, O 10%	1.60	(1.40‐1.78)	(1.00‐3.40)	0.0%	0.7%
	T 20%, O 10%	1.29	(1.00‐1.50)	(0.89‐2.25)	0.0%	0.0%
Nearest Neighbor Caliper Matching	T 50%, O 50%	1.57	(1.50‐1.67)	(1.27‐2.06)	0.0%	2.7%
	T 20%, O 50%	#N/A	#N/A	#N/A	0.0%	0.0%
	T 50%, O 10%	1.60	(1.45‐1.70)	(1.09‐2.11)	0.0%	0.0%
	T 20%, O 10%	#N/A	#N/A	#N/A	0.0%	0.0%

*Note:* The calliper was set at 0.01 of the standard deviation of the propensity score. The % sign low and sign high risk state in how many of the successfully matched cohorts the treatment‐outcome associations was statistically different from 1.

Abbreviation: OR, odds ratio.

### A..4 References

1. Forslund T, Wettermark B, Wändell P, et al. Risk scoring and thromboprophylactic treatment of patients with atrial fibrillation with and without access to primary healthcare data: Experience from the Stockholm health care system. Int. J. Cardiol. 2013;170 (2):208–214.

2. Komen JJ, Hjemdahl P, Mantel‐Teeuwisse AK, et al. Concomitant Anticoagulant and Antidepressant Therapy in Atrial Fibrillation Patients and Risk of Stroke and Bleeding. Clin. Pharmacol. Ther. 2019;cpt.1603.

3. Lip GYH, Nieuwlaat R, Pisters R, et al. Refining clinical risk stratification for predicting stroke and thromboembolism in atrial fibrillation using a novel risk factor‐based approach: the euro heart survey on atrial fibrillation. Chest. 2010;137 (2):263–72.
